# The Impact of Intraoperative Haemodynamic Monitoring, Prediction of Hypotension and Goal-Directed Therapy on the Outcomes of Patients Treated with Posterior Fusion Due to Adolescent Idiopathic Scoliosis

**DOI:** 10.3390/jcm12144571

**Published:** 2023-07-09

**Authors:** Agata Andrzejewska, Jakub Miegoń, Sławomir Zacha, Karolina Skonieczna-Żydecka, Konrad Jarosz, Wojciech Zacha, Jowita Biernawska

**Affiliations:** 1 Department of Anaesthesiology and Intensive Care, Pomeranian Medical University, 71-252 Szczecin, Poland; 2 Department of Paediatric Orthopaedics and Oncology of the Musculoskeletal System, Pomeranian Medical University, 71-252 Szczecin, Poland; 3 Department of Biochemical Science, Pomeranian Medical University, 71-460 Szczecin, Poland; 4 Department of Clinical Nursing, Pomeranian Medical University, 71-210 Szczecin, Poland

**Keywords:** adolescent idiopathic scoliosis, haemodynamic monitoring, hypotension prediction index, ERAS (enhanced recovery after surgery)

## Abstract

A prospective, single-centre, non-randomised, case–control study aimed to evaluate the effectiveness of intraoperative haemodynamic monitoring, prediction of hypotension and goal-directed therapy on the outcomes of patients undergoing posterior fusion for adolescent idiopathic scoliosis (AIS). The control group (*n* = 35, mean age: 15 years) received standard blood pressure control during surgery, while the intervention group (*n* = 24, mean age: 14 years) underwent minimally invasive haemodynamic monitoring and goal-directed therapy. The intervention group showed significantly shorter durations of hypotension (mean arterial pressure < 60 mmHg), reduced hospital stays and smaller decreases in post-surgery haemoglobin levels. Additionally, the intervention group experienced shorter times from the end of surgery to extubation. These findings suggest that incorporating targeted interventions during intraoperative care for AIS patients undergoing posterior fusion can lead to improved outcomes.

## 1. Introduction

Adolescent idiopathic scoliosis (AIS) is the most common spinal deformity in children. The most commonly occurring (in 80%) idiopathic scoliosis is a three-dimensional spinal deformity, and the exact cause of which is not yet fully understood. Ongoing research into the aetiology of this condition suggests possible genetic factors. Idiopathic scoliosis is diagnosed when the curvature exceeds 10 degrees. Approximately 2–3% of the entire population is affected by this deformity. It occurs more frequently in children and adolescents from families where this condition has already been present (around 7%). Surgical treatment is indicated when the spinal curvature reaches approximately 40–50 degrees. Surgical correction is a challenge for the anaesthetist because of the large surgical area and the need for specific anaesthetic techniques adapted to intraoperative neurophysiological monitoring of the spinal cord. The risk of spinal cord injury can be reduced by using intraoperative neuromonitoring and maintaining adequate spinal cord perfusion.

Intraoperative haemodynamic stability remains an important factor in the post-operative prognosis of patients undergoing surgery for AIS. Hypotension and consequent tissue hypoperfusion are major causes of poor neurological outcomes. Monitoring intraoperative volemia, cardiac function, blood pressure and haemoglobin level helps to ensure adequate oxygen delivery (DO) to the organs. Tissue hypoxia is one of the major causes of perioperative complications [1]. Appropriate fluid and vasopressor management helps to prevent and treat hypo- and hypervolemia and is necessary to maintain adequate DO while avoiding fluid overload.

Intraoperative hypotension (IOH) is common during surgery. There is no universally accepted definition of IOH. Different definitions describe different incidences. IOH has the potential to cause ischaemia–reperfusion injury, which may manifest as dysfunction of any vital organ [2]. There are some studies reporting its association with post-operative complications in adults, but few in children. In APRICOT, hypotension was reported in 54.9% of all major cardiovascular events. However, the majority (94%) of these events had an uneventful outcome [3].

Standard methods based on non-invasive blood pressure monitoring may not be sufficient to detect perfusion abnormalities. Blood pressure may be normal during hypovolemia due to increased vascular resistance; heart rate may not reflect hypovolemia due to the influence of anaesthesia. What is more important with oscillometric technique, in relation to the invasive blood pressure measurement, is it overestimates low blood pressure readings and underestimates high blood pressure readings. Therefore, more advanced haemodynamic monitoring methods and devices have been developed.

Uncalibrated devices use the patient’s anthropometric and demographic characteristics, internal databases and algorithms to calculate the cardiac output from the arterial waveform, which is suitable for perioperative optimisation protocols. Calculated, measured or derived haemodynamic parameters are useful in determining the causes of hypotension. The ability to predict intraoperative hypotension allows a shift from reactive to proactive management. The cause of impending IOH can be identified earlier and appropriate treatment can be initiated sooner. The duration of hypotension can be reduced and hypotension-related complications can be effectively prevented. This idea is now available in practice. An Edwards Lifesciences machine learning algorithm—Acumen Hypotension Prediction Index (HPI)—based on thousands of arterial waveform features, can identify a hypotensive event 15 min before it occurs with high sensitivity and specificity. Currently, there are no other reliable methods of predicting the likelihood of hypotension.

Goal-directed therapy (GDT) is a haemodynamic treatment based on the titration of fluid and inotropic agents infused to physiological flow-related endpoints [4]. Implementation of GDT strategies leads to a reduction in perioperative morbidity and mortality in both adults and children [5,6,7,8,9,10,11,12]. However, the transfer of monitoring methods developed primarily for adults to children can lead to pitfalls due to the different characteristics of the vascular system [13] and the lack of reference values [14].

Advanced haemodynamic parameters routinely used in adults have a poor correlation in children [13]. No arterial pressure or plethysmographically derived variable is accurate in predicting fluid responsiveness in children. This poor correlation with adults is probably due to greater vascular compliance. However, the age at which arterial properties in children become similar to those in adults is not known. Further research is needed. This article is the first step towards validation of the method in children.

The aim of this study was to evaluate the effectiveness of intraoperative haemodynamic monitoring, prediction of hypotension and targeted therapy on the outcome of patients treated with posterior fusion for AIS.

## 2. Materials and Methods

A prospective, single-centre, non-randomised, case–control study was conducted. The study included 59 consecutive Caucasian teenagers undergoing posterior fusion due to adolescent idiopathic scoliosis in the Department of Paediatric Orthopaedics and Oncology of the Musculoskeletal System, Pomeranian Medical University, in Szczecin, Poland. The inclusion criteria were first scoliosis operation and age under 18 years. Patients who were qualified for scoliosis surgery in this centre underwent X-ray imaging, computed tomography and magnetic resonance imaging. Patients with a Cobb angle greater than 45 degrees were considered eligible. Prior to anaesthesia qualification, echocardiography and spirometry examinations were performed. The exclusion criteria were advanced chronic respiratory–circulatory failure, emergency surgery, reoperation and scoliosis type other than idiopathic.

**Figure 1:**
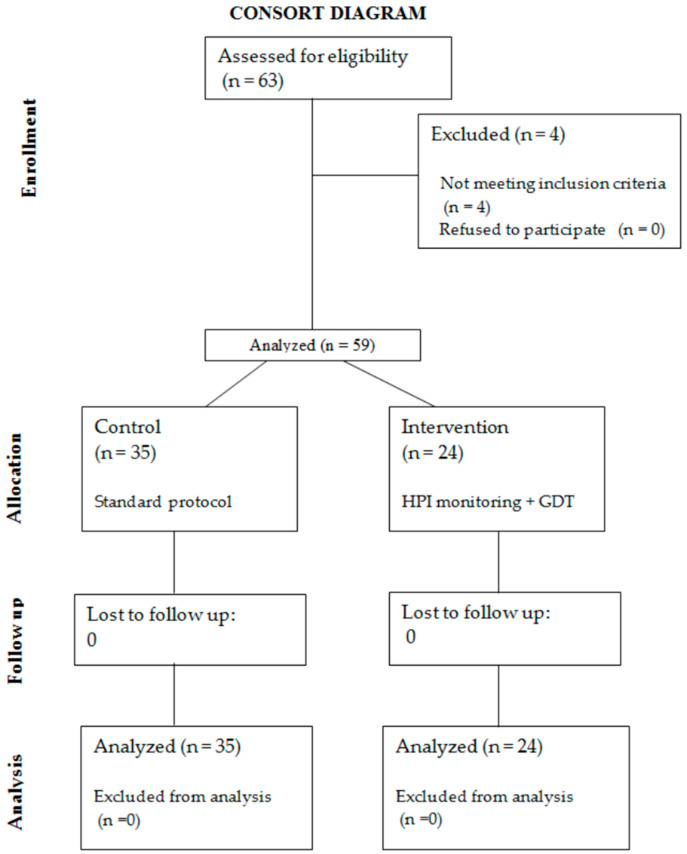
The Consort diagram.

The “control group” consisted of 35 patients (mean age 15 years), among whom 7 were boys, treated with a standard protocol of blood pressure management during surgery. The results were analysed, and based on these, the protocol of the “intervention group” was established. The “intervention group” consisted of 24 patients (mean age 14 years), with a predominance of females (3 boys). In the latter group, intraoperative minimally invasive haemodynamic monitoring and goal-directed therapy were adopted. The Consort diagram provided in Figure 1 illustrates the flow of participants throughout the study.

The study protocol was registered at clinicaltrials.gov. under the accession no. NCT 05159505.

### 2.1. Preparation for Surgery

After the surgery was scheduled, the patients were referred for an anaesthetic consultation and then qualified for general anaesthesia.

On the day of the procedure, as a pre-emptive analgesia, the patient received metamizole (15 mg/kg for children under 50 kg or 1 g orally if the body weight exceeds 50 kg), along with a carbohydrate-rich fluid (Preop, Nutricia Poland) administered orally.

### 2.2. Surgery

**Figure 2:**
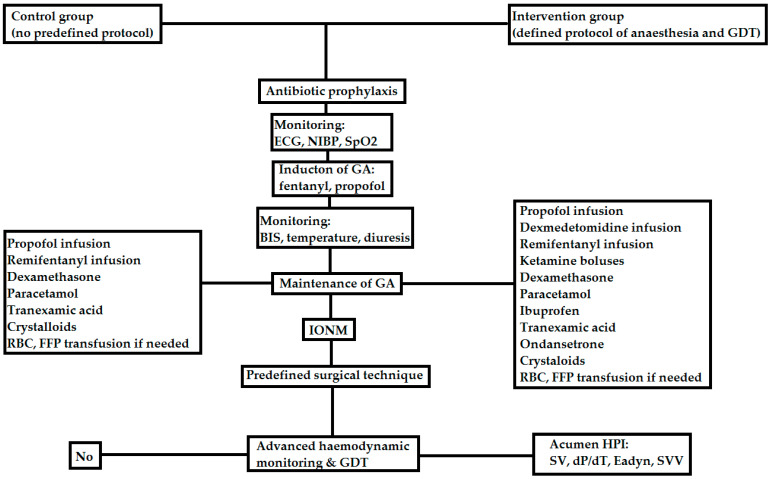
The anaesthesia protocol used in this study (ECG—electrocardiography, NIBP—non-invasive blood pressure, SpO2—pulse oximetry, GA—general anaesthesia, RBC—red blood cells, FFP—fresh frozen plasma, IONM—intraoperative neuromonitoring, HPI—hypotension prediction index, dP/dT—systolic slope, Eadyn—effective arterial elastance, SVV—stroke volume variation).

All patients underwent general anaesthesia with intubation with a reinforced endotracheal tube and mechanical ventilation with a Primus apparatus (Dräger, Lübeck, Germany). The control group did not follow a defined anaesthesia protocol. The administration of anaesthesia in the intervention group is depicted in Figure 2.

**Figure 3:**
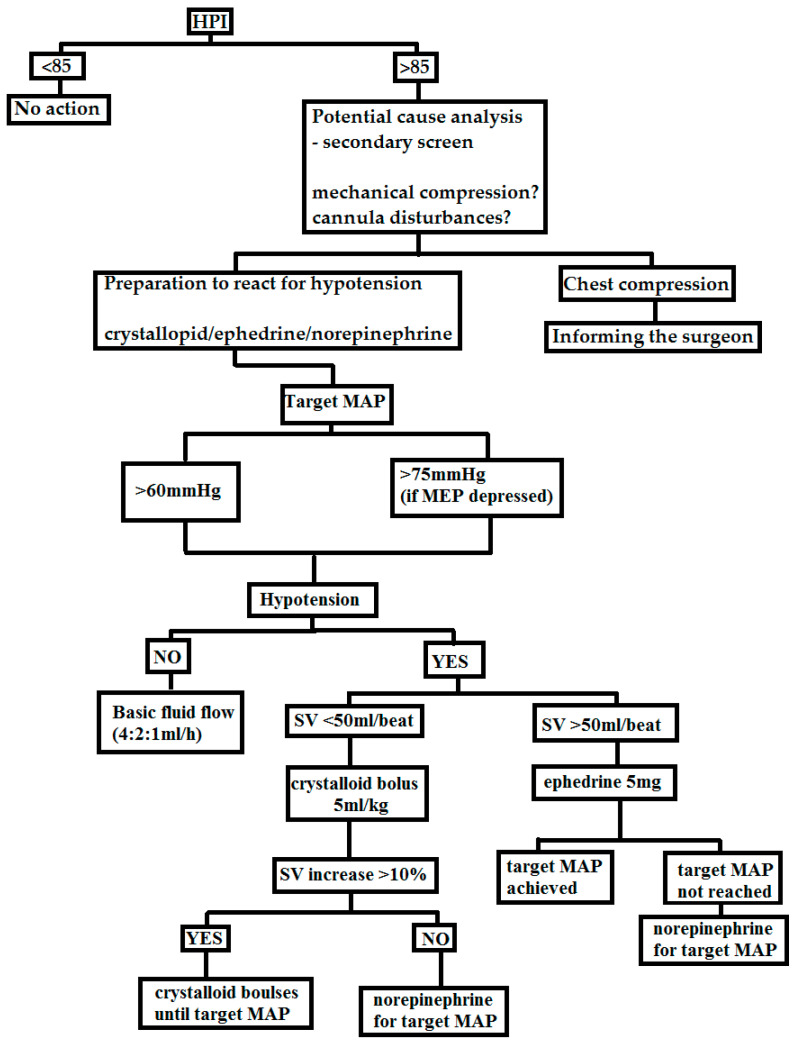
The GDT protocol used in this study.

The goal-directed therapy (GDT) protocol used in this study consisted of maintaining the desired levels of mean arterial pressure (MAP) and stroke volume (SV) through fluid therapy and vasopressor administration. The protocol is presented in Figure 3.

Fluid therapy consisted of balanced crystalloid (Sterofundin—B. Braun/Optilyte—Fresenius Kabi) given at a rate of 4:2:1 mL/kg intravenously. The protocol was initiated when the patient was placed in the prone position, prior to which a bolus of 5 mg IV ephedrine was administered in response to hypotension. The target values were MAP > 60 mmHg, and in the case of depressed motor-evoked potentials (MEPs), MAP was maintained at >75 mmHg. If hypotension (MAP < 60 mmHg) and SV < 50 mL/beat were observed, a bolus of crystalloid at a dose of 5 mL/kg IV was given over 10–15 min. After the bolus, the response to fluid therapy was assessed. If SV increased by >10% and hypotension persisted, another fluid bolus was given. If there was no increase in SV > 10%, norepinephrine infusion was started to maintain MAP > 60 mmHg. If hypotension (MAP < 60 mmHg) was observed and SV was >50 mL/beat, a single dose of 5 mg ephedrine was administered. If the response was inadequate, norepinephrine infusion was started to maintain the target MAP. If SV was <50/beat but without evidence of hypotension (MAP > 60 mmHg) or peripheral perfusion failure—capillary refill time (CRT) < 2 s, no fluid bolus was administered. Colloid administration was prohibited. If blood loss exceeded 7 mL/kg, 1 unit of red blood cells was transfused.

In this study, the intervention arm consisted of patients monitored with the Edwards Lifesciences Hypotension Prediction Index (HPI) device, specifically the Acumen sensor and Hemosphere monitor. When the HPI device’s hypotension alarm (HPI > 85) was triggered in the intervention group, the anaesthesiologist responded immediately by using a secondary screen. This screen allowed the assessment of the patient’s stroke volume variation (SVV), systolic slope/contractility (dP/dT) and dynamic arterial elastance (Eadyn) and made treatment decisions accordingly. The anaesthesiologist administered fluid boluses or vasopressors based on the patient’s specific condition and the secondary screen readings. This personalised approach to treatment prevented further hypotensive events [15].

### 2.3. Surgical Technique

The surgical technique consisted of a posterior approach, ligament and bone release, implant fixations and final correction on titanium rods. The procedures were performed using a screws-only system or hybrid systems (screws, hooks or sublaminar bands). The estimated blood loss for this surgical procedure is over 7 mL/kg. Patients are prepared with 2 units of packed red blood cells. The procedures are performed by two independent operators working simultaneously, utilising two electrocautery instruments concurrently. This approach reduces the surgical time and minimises blood loss.

### 2.4. Intraoperative Neuromonitoring (IONM)

Throughout the procedure, the evoked motor and sensory potentials are monitored in order to control the function of the spinal cord. All screws were checked using direct nerve stimulation (DNS) electromyography.

### 2.5. Post-Operative Course

After surgery, all patients were monitored in the post-anaesthesia care unit (PACU) for 24 h. In addition to the assessment of vital signs, a protocol for the assessment of pain intensity according to the numerical scale (NRS) was performed every hour for 24 h and then every 8 h until discharge from the hospital, including the type, dose and route of administered drugs, the occurrence of side effects and complications. In the post-operative period, drugs were administered at regular intervals: paracetamol, metamizole, ibuprofen, magnesium sulphate, morphine and lignocaine infusions. Intravenous opioid infusion and transition to oral treatment were determined according to daily requirements.

The described extent of preoperative preparation, the surgical technique, the type of analgesia used in the perioperative period and the parameters evaluated after surgery were the same in the “control” and “intervention” groups. In both groups, the hospitalisation plan was based on the principles of the enhanced recovery after surgery (ERAS) protocol. The current definition of ERAS programmes involves a multidisciplinary approach to improving surgical outcomes through the use of procedure-specific, evidence-based protocols in the care of surgical patients.

### 2.6. Definitions of Complications

Hypotension was defined as systolic blood pressure (SBP) less than 90 mmHg and MAP less than 60 mmHg for at least 1 min.

Adverse drug reactions were nausea, vomiting, pruritus, dyspnoea, constipation, urinary retention, dizziness, drowsiness preventing rehabilitation, apnoea, hypotension, bradycardia and decrease in peripheral capillary oxygen saturation (SpO_2_) < 90%.

Surgical complications were transient neuropraxia related to positioning, partial or complete spinal cord injury with resulting transient or fixed paralysis, dural tear, visual disturbances, position-related complications, respiratory–circulatory failure, haematoma, surgical site infection, pneumonia, gastric disorders and death.

The evaluation of the obtained results was a comparison in terms of demographic data, total time of hypotension, duration of the surgical procedure “skin to skin” (minutes) and duration of hospitalisation (days).

### 2.7. Outcomes

The primary outcome was intraoperative hypotension time. The secondary outcomes were as follows:

- Duration of the surgical procedure “skin to skin” (minutes)
- Duration of hospitalisation (days)
- Neurological complications (no)
- Cardiac complications (no)
- Red blood cells transfusion volume (mL)
- Fresh frozen plasma transfusion volume (mL)
- Intraoperative blood loss (mL)
- Crystalloids administered (mL/kg)
- Ephedrine total dose (mg)
- Haemoglobin level before surgery (g/dL)
- Haemoglobin level after surgery (g/dL)
- Change in haemoglobin level (g/dL)
- Haematocrit level before surgery (%)
- Haematocrit level after surgery (%)
- Change in haematocrit level (%)
- Time to extubation (min)

### 2.8. Statistical Analyses

The Shapiro–Wilk normality test was carried out to assess the distribution of continuous variables. Descriptive statistics were consequently presented as means and standard deviations or median and interquartile ranges (IQRs) as appropriate. Qualitative variables were presented as numbers and percentages. To compare the outcomes between the two groups, non-parametric Mann–Whitney or *t*-test was used as appropriate in regard to Shapiro–Wilk test. To analyse the link between qualitative variables, the chi^2^ test was used. The acceptable probability of error for the first type was assumed to be equal to 0.05. MedCalc statistical software version 20.110 (Ostend, Belgium) was used. For controlling type I errors, the false discovery rate (FDR) approach was used. The calculations were performed using the p.adjust function of the stats package in R [16]. Statistical power was calculated using G*Power software version 3.1.9.2 [17].

## 3. Results

Baseline characteristics of the study participants in terms of the tested procedure in Table 1. No significant differences were found between any of the tested parameters.

Table 2 provides a concise overview of a comparative analysis conducted in perioperative data, hypotension time, transfusions and haemoglobin levels in the control and intervention groups. It demonstrates that the intraoperative haemodynamic monitoring significantly affected the time of hypotension, duration of hospital stay, fresh frozen plasma (FFP) transfusion, time to extubation, intraoperative diuresis and almost all but one of the tested blood parameters. The duration of hypotension with MAP below 55 mmHg was found to be minimal in the intervention group, with a median of 0 min and an IQR of 0–2 min. The intervention group did not require the utilisation of norepinephrine throughout the study. The mean duration of SVV above 12% was 82 ± 59.7 min. After correction for multiple testing, a statistical significance changed into tendency (*p* = 0.06) in regard to intraoperative diuresis.

**Figure 4:**
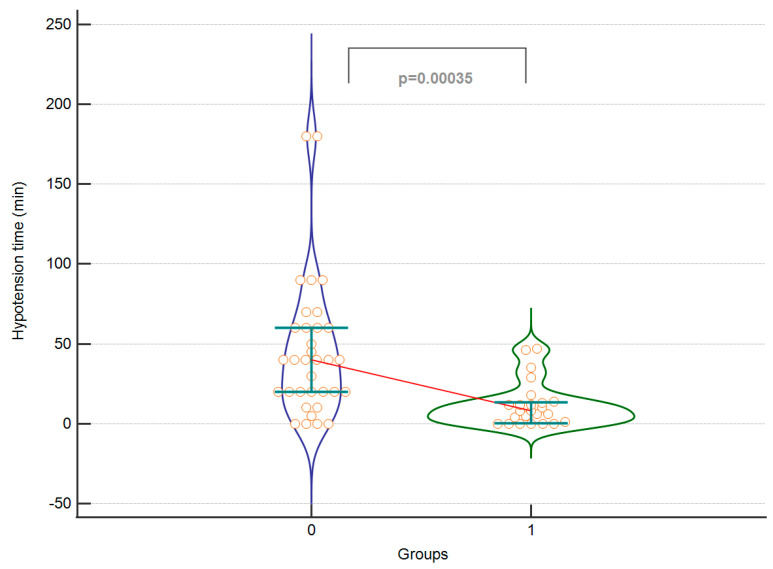
The perioperative hypotension time between the control (0) and the intervention (1) groups. A violin plot depicting medians and IQRs. Orange circles represent individual cases. The red horizontal line connects the medians.

**Figure 5:**
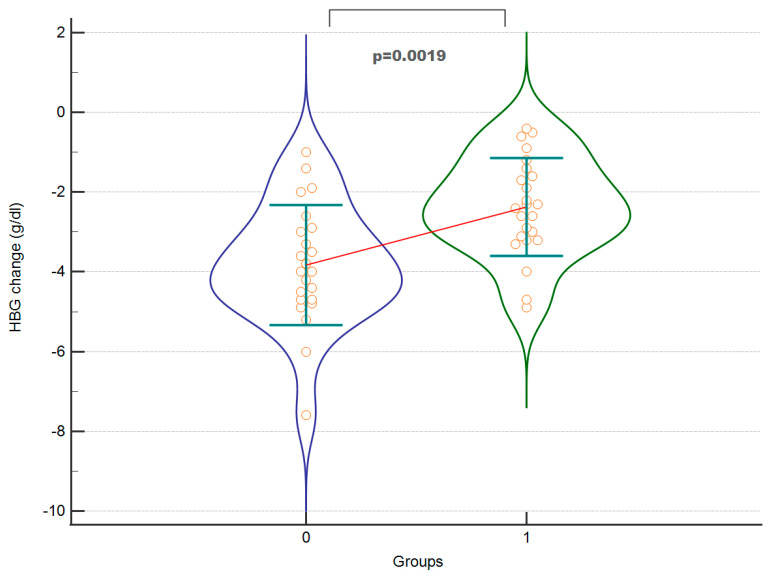
The change in haemoglobin level (endpoint-baseline value) between the control (0) and the intervention (1) groups. A violin plot depicting means and SD. Orange circles represent individual cases. The red horizontal line connects the means.

Exemplary Figure 4 and Figure 5 of significant results are presented below.

There were four complications of neurological and cardiac nature in the control group alone (cardiorespiratory failure due to hypovolemic shock—three patients and transient limbs paresis—one patient). No such events occurred in subjects from the intervention group.

## 4. Discussion

The present study is the first report to evaluate the effectiveness of intraoperative haemodynamic monitoring, prediction of hypotension and targeted therapy on outcomes in adolescents undergoing posterior fusion for AIS.

The study showed a significantly shorter duration of hypotension (MAP < 60 mmHg) in patients in the intervention group. There were no differences in the duration of surgery between the two groups, probably due to the same operators. The length of hospital stay was significantly shorter in the intervention group. In the control group, there were instances of cardiac and neurological complications, while no such complications were observed in the intervention group following the GDT protocol. Apart from these specific complications, no other adverse events were observed in either the control or intervention groups. The amount of crystalloids administered and the total dose of ephedrine were similar in both groups. Blood loss was similar in both groups, although fresh frozen plasma was transfused more frequently in the control group. There was a significantly smaller decrease in haemoglobin levels after surgery and a trend towards a smaller difference in haematocrit in the intervention group, despite higher baseline values in the control group. The time from the end of surgery to extubation was significantly shorter in the intervention group. Intraoperative diuresis was significantly higher in the intervention group.

These positive results suggest that the intervention has the potential to improve post-operative recovery and overall patient outcomes.

Spinal deformity surgery carries a risk of spinal cord injury of 0.3% to 0.6% according to surveys conducted by the Scoliosis Research Society [18]. These neurological injuries can result from direct compression of the spinal cord by implants or surgical instruments, reduction of blood flow to the spinal cord by stretching or compression of vessels or direct interruption of radicular blood flow, distraction injury to the spinal cord or epidural haematoma. Of these, ischaemic injury is the most common. The motor pathways supplied by the anterior spinal artery are the most vulnerable areas of the spinal cord to ischaemic injury. In this context, the ability to detect and prevent hypotension during spinal deformity surgery is critical to the prevention of ischaemic injury. MAP below 60 mmHg during spinal surgery has been shown to be a critical risk factor for spinal cord injury [19]. However, early detection of hypotension can be challenging, particularly in children, due to the limited availability of non-invasive monitoring options.

The use of HPI monitoring, therefore, may offer a potential solution to this problem, as it has been shown to accurately predict hypotension in adults. In this study, the authors aim to evaluate the potential benefits of HPI monitoring in identifying patients at risk of hypotension during scoliosis surgery, with a particular focus on the prevention of intraoperative spinal cord ischaemia, the most common complication. The results of the present study indicate that invasive arterial pressure monitoring, together with the use of the HPI tool and the GDT protocol, allows a significant reduction in the duration of intraoperative hypotension. Moreover, it was observed that the amount of time during which the MAP remained below 55 mmHg was extremely brief. There is no existing research on the use of HPI monitoring in children, highlighting the need for further investigation into its potential benefits and applicability in this population.

The Edwards Lifesciences HPI is a new technology that aims to predict the onset of hypotension before it occurs. The HPI is designed to identify potential hypotensive events in the next 5 to 15 min.

Artificial intelligence (AI) is becoming an increasingly important part of medicine, with its use expanding every year. AI can be defined as the study of algorithms that give machines the ability to reason and perform functions such as problem-solving, object and word recognition, inference of world states and decision-making.

The HPI is a supervised machine learning (ML) algorithm, a branch of AI based on the idea that a computer can be trained on specific input data and apply the knowledge gained from that data to newly presented data without being explicitly programmed to do so. This means that it has been trained to classify labelled outputs in order to predict a desired or undesired event. Supervised ML algorithms are trained on a set of labelled data (i.e., the training set), and then their predictive accuracy is tested on new data. The HPI algorithm went through internal and external validation sets, ultimately resulting in a highly accurate algorithm with an area under the receiver operating characteristic curve (AUC) of 0.95, 0.95 and 0.97 for 15, 10 and 5 min before a hypotensive event, respectively.

The HPI algorithm uses several parameters derived from the arterial pressure waveform to predict the onset of hypotension. SV is the amount of blood pumped by the heart in one heartbeat. SVV is the percentage change in stroke volume during mechanical ventilation. These two parameters are derived from the arterial pressure waveform. Effective arterial elastance (Eadyn) is a measure of the ability of the arterial system to change size and shape in response to blood flow. It is calculated by dividing pulse pressure variation (PPV) by SVV. PPV is a measure of the variation in pulse pressure waveform during mechanical ventilation, while SVV is the variation in stroke volume during the same period. By analysing Eadyn, clinicians can determine whether the patient is likely to respond to additional fluids to raise blood pressure. In other words, Eadyn provides an indication of afterload responsiveness, or the ability of the arterial system to raise blood pressure by increasing the volume of blood pumped by the heart. dP/dt is a measure of the rate at which the arterial pressure waveform rises during systole. It is calculated by measuring the maximum slope of the arterial pressure waveform from a peripheral artery. The dP/dt values provide clinicians with information about the contractility of the heart. Using these parameters derived from the arterial pressure waveform, clinicians can predict the onset of hypotension and choose an appropriate intervention to prevent it. For example, if the HPI algorithm predicts that a patient is at risk of hypotension due to low stroke volume, clinicians can choose to administer fluids to increase preload and maintain blood pressure. Alternatively, if the patient’s afterload responsiveness is low, clinicians may choose to administer vasopressors to increase vascular resistance and maintain blood pressure. By using these parameters to guide decision-making, clinicians can take timely preventative action and avoid the potentially harmful consequences of hypotension.

While the HPI is a useful tool for predicting the onset of hypotension, it has some limitations that clinicians should consider. The HPI algorithm has been developed and validated using data from specific patient populations and clinical settings. The algorithm was developed using records from operating theatre and intensive care unit patients and validated using a physiological dataset from surgical patients. While the HPI algorithm has shown promising results in these populations, its performance may vary in different patient populations or clinical contexts. In addition, clinicians must also consider the potential cardiodepressive effects of intravenous anaesthetic induction when assessing the risk of hypotension. Induction of anaesthesia can cause hypotension and bradycardia due to depression of cardiac contractility and peripheral vascular resistance. The index predicts hypotension but does not prevent hypotension in the absence of treatment. Without clinician awareness of the importance of hypotension prevention and immediate treatment, the protocol will fail. Education and confidence in new tools are very important parts of success in hypotension prevention.

Although HPI monitoring has not been validated in the paediatric population and is not recommended by Edwards Lifesciences, the authors decided to use it in adolescents undergoing scoliosis surgery. This decision was based on the similar body surface area of the adolescents in our study to the patients in the HPI validation study [20]. While the HPI algorithm is derived from the Edwards Lifesciences Flotrac arterial-pressure-based cardiac output (APCO) monitoring system, which has been shown to be useful in the paediatric population, Flotrac itself has not been validated in children. The authors believe that this study can serve as a first step towards the validation of HPI monitoring in adolescents and may provide insights into the applicability of HPI monitoring in children. The limited research into the use of HPI monitoring in children highlights the need for further research into its potential benefits and applicability in this population.

GDT protocols have become increasingly popular in the operating theatre as a way to optimise haemodynamic status and improve patient outcomes. There are several types of GDT protocols that can be used, including optimisation of stroke volume, cardiac output and oxygen delivery. These protocols involve the use of advanced monitoring techniques, such as arterial waveform analysis, echocardiography and pulse contour analysis, to guide fluid and vasopressor administration to achieve specific haemodynamic targets. Other protocols may be based on the use of dynamic variables such as SVV or PPV to guide fluid management. The use of GDT has been shown to improve outcomes in a range of surgical procedures, including high-risk surgery and major abdominal surgery. In the recently updated European Society of Cardiology (ESC) guidelines on cardiovascular assessment and management of patients undergoing non-cardiac surgery, perioperative goal-directed haemodynamic therapy in patients undergoing high-risk surgery received a Class IA recommendation. In addition, there is a strong recommendation (IB) to avoid an intraoperative decrease in MAP > 20% from baseline or below 60–70 mmHg to avoid perioperative complications [21].

Although GDT protocols are well established in the adult population, there is a paucity of literature on their use in children. A study by Pereira de Souza Neto et al. [13] investigated the use of dynamic parameters and transthoracic echocardiography to predict fluid responsiveness in mechanically ventilated children under general anaesthesia. The study found that although the respiratory variation of aortic peak velocity (△Vpeak) was an accurate predictor of fluid responsiveness, no arterial pressure or plethysmographically derived variable was accurate in predicting fluid responsiveness. However, Koraki et al. used an SVV and ClearSight (Edwards Lifesciences) based GDT protocol with fluid boluses and norepinephrine in scoliosis surgery [12]. In their single-centre retrospective analysis, the authors found that the SVV and ClearSight-based GDT protocol was effective in maintaining haemodynamic stability and achieving favourable outcomes in scoliosis surgery patients. Specifically, they observed that the protocol was associated with a reduced need for blood transfusions, shorter hospital stays and lower rates of post-operative complications. These findings suggest that GDT protocols may be beneficial in paediatric populations undergoing surgery, particularly those undergoing high-risk procedures, such as scoliosis correction. Further research is needed to determine the optimal approach to GDT in children and to identify the most effective protocols to improve outcomes in this population.

The decision to use an arterial waveform analysis GDT protocol rather than pulse contour analysis or ultrasound analysis is based on several factors. First, arterial waveform analysis is a well-established and validated method for assessing fluid responsiveness and provides continuous and real-time feedback on haemodynamic changes. In contrast, echocardiographic analysis requires specialised training and may not be feasible in all clinical settings; it is typically performed at discrete time points and may not be able to detect acute changes in haemodynamic status. Non-invasive pulse contour analysis may be affected by factors such as hypothermia, vasoconstriction or centralisation of the circulation, which may alter the accuracy of the technique.

The authors chose to implement a stroke-volume-based GDT protocol. Alternative SVV-based protocols can be problematic in clinical situations where there are changes in intrathoracic pressure, such as during scoliosis correction. This is because SVV is affected by changes in venous return and compliance, which can lead to misinterpretation and inappropriate fluid management decisions. Secondly, protocols based on cardiac output (CO) are more prone to variability due to changes in heart rate, which can lead to inaccurate measurements, making them less reliable.

It is worth noting that the validation study of the HPI algorithm did not include hypotensive events caused by clinical interventions, such as laparoscopic insufflation, liver manipulation or vascular clamping [22].

The algorithm requires a “clean” arterial waveform. A fast flush test should be performed at the beginning of each procedure to ensure that the waveform is acceptable.

It is crucial to recognise that during scoliosis correction procedures, the anaesthetist should not blindly follow the conventional GDT protocol and should stay aware of its limitations during this type of surgery. This is due to the fact that there are moments during the procedure when the surgeon applies a significant amount of force, which can lead to thoracic compression and kinking of the arterial cannula. As a result, the arterial pressure waveform may change to a straight line, which the HPI software may interpret as hypotension. This can cause ambiguity as the typical response to hypotension is to initiate the GDT protocol. However, in the context of scoliosis correction procedures, it is essential to recognise that the appropriate action is to reduce the force exerted by the operator on the surgical instruments. By adjusting the level of force applied, it is possible to avoid hypotension and maintain haemodynamic stability in the patient. This approach is essential to ensure that patient outcomes are optimised and problems are avoided. Failure to adjust treatment plans in response to real-time physiological changes can lead to several adverse outcomes, including increased morbidity and mortality, prolonged hospital stays and increased procedural costs. Due to the complex and multifaceted nature of scoliosis correction, it is essential for the surgical team to remain vigilant and adaptable throughout the procedure. By closely monitoring the patient’s physiological responses and responding appropriately to changes in the haemodynamic profile, the surgical team can ensure that the patient receives the highest standard of care and achieves optimal outcomes.

Despite the promising findings, there are several limitations to this study. Firstly, the study design was a prospective, single-centre, non-randomised, case–control study, which may limit the generalisability of the results. The lack of randomisation and inclusion of patients from a single centre introduce the possibility of selection bias and may not accurately represent the wider population of patients with adolescent idiopathic scoliosis undergoing posterior fusion. Also, the lack of blinding in the intervention group introduces the potential for performance bias, as clinicians were aware of the treatment they were giving. Finally, there is a concern that overfitted models will not replicate in future studies, which gives the impression of uncertainty about the scientific value of the discovery. Of course, this is also a possibility in this study, mainly due to the relatively small size of the study groups. However, the multiple comparisons made by the authors and the statistical power higher than acceptable in scientific research allow for the hope that the results will be reflected in groups of larger size and for other clinical diagnoses and, consequently, surgical procedures. Therefore, the authors recommend a degree of caution when interpreting the results of our study.

Despite these limitations, this study provides valuable insights into the potential benefits of intraoperative haemodynamic monitoring and goal-directed therapy in patients with adolescent idiopathic scoliosis and warrants further investigation in larger, multicentre, randomised controlled trials.

In conclusion, protocolised haemodynamic monitoring and fluid management have emerged as essential components of the paediatric ERAS protocol. Combined with intraoperative neuromonitoring, this approach has the potential to significantly reduce hospital stay, post-operative complications such as neurological injury, blood loss and wound infection. Recent studies have shown that the implementation of haemodynamic optimisation and ERAS protocols can lead to improved outcomes in high-risk surgical procedures, such as scoliosis surgery. The use of real-time haemodynamic monitoring and goal-directed therapy protocols can help ensure that patients receive individualised care tailored to their specific physiological needs.

As research in this area continues, it is important to continue to evaluate and compare different haemodynamic monitoring and fluid management protocols to determine which approach is most effective in improving outcomes and reducing complications in paediatric surgical patients. Further studies are needed to refine the protocol and identify best practice guidelines for scoliosis surgery to optimise patient outcomes and reduce the healthcare burden associated with this complex procedure.

## 5. Conclusions

In conclusion, the implementation of intraoperative haemodynamic monitoring, prediction of hypotension and goal-directed therapy in patients undergoing posterior fusion for adolescent idiopathic scoliosis confirmed shorter intraoperative hypotension time, reduced length of hospitalization and enhanced preservation of haemoglobin levels. Further studies and wider implementation of these strategies are warranted to fully assess their impact and establish their effectiveness as standard practice in this patient population.

## Figures and Tables

**Table 1:** Demographic data from patients among the groups.

Parameters	Control (*n* = 35)	Intervention (*n* = 24)	*p*
Age	14 (13–15)	15 (14–16)	0.092
Gender (boys/girls)	7/28	3/21	0.559
BMI *	20.58 (18.43–22.37)	19.86 (18.43–21.82)	0.635
Haemoglobin level before surgery (g/dL)	13.71 (13.00–14.60) *	13.02 (12.25–14.10) *	0.041
Haematocrit level before surgery (%)	38.29 (37.00–40.00) **	37.83 (35.10–40.30) **	0.637
ASA * 1	21	15	0.439
ASA 2	14	8	
ASA > 2	0	1	

Legend: BMI—body mass index, ASA—American Society of Anaesthesiology; * median, interquartile range; ** mean, interquartile range.

**Table 2:** Perioperative data, time of hypotension, complications and transfusions.

Parameters	Control (*n* = 35)	Intervention (*n* = 25)	*p*	FDR	Power
Duration of the surgical procedure “skin to skin” (min)	195 (166–218) *	200 (170–240) *	0.542	0.580	0.99
Hypotension time (min)	40 (20–60) *	8 (0.5–13.5) *	<0.001	0.003	0.98
Duration of hospitalisation (days)	10 (9–11) *	7 (6–8) *	<0.001	0.003	0.99
Red blood cells transfusion volume (mL)	290 (0–560) *	290 (0–290) *	0.230	0.335	0.23
Fresh frozen plasma transfusion volume (mL)	200 (0–200) *	0 *	<0.001	0.003	0.99
Intraoperative blood loss (mL)	500 (350–500) *	500 (375–575) *	0.816	0.816	0.11
Intraoperative blood loss (mL/kg)	7 (5–10) *	5.55 (0–9.5) *	0.246	0.336	0.62
Crystalloids administered (mL/kg)	22 (16.3–31.5) *	24.2 (19.8–31.8) *	0.430	0.496	0.25
Ephedrine total dose (mg)	0 (0–8.75) *	5 (0–7.5) *	0.322	0.402	0.99
Haemoglobin level after surgery (g/dL)	9.71 (1.59) **	10.65 (1.05) **	0.020	0.043	0.71
Change in haemoglobin ^ (g/dL)	−3.83 (1.50) **	−2.3708 (1.22) **	<0.001	0.003	0.29
Haematocrit level after surgery (%)	27.93 (4.05) **	30.85 (3.24) **	0.008	0.002	0.82
Change in haematocrit level ^ (%)	−9.53 (5.39) **	−6.98 (3.69) **	0.063	0.105	0.51
Time to extubation ^$^ (min)	27.5 (20–50) *	5 (0–17.5) *	<0.001	0.003	0.98
Intraoperative diuresis (mL)	135 (100–250)	215.5 (129–300)	0.037	0.069	0.18

Legend: * median, interquartile range; ** mean, SD; ^ before–after surgery; ^$^ time from the end of operation till extubation.

## Data Availability

The raw data are available upon request from the corresponding author.
